# Analytical and clinical performance of a Chikungunya qRT-PCR for Central and South America^☆^^☆☆^

**DOI:** 10.1016/j.diagmicrobio.2017.06.001

**Published:** 2017-09

**Authors:** Thomas Edwards, Leticia del Carmen Castillo Signor, Christopher Williams, Clément Larcher, Mauricio Espinel, Jane Theaker, Evelin Donis, Luis E. Cuevas, Emily R. Adams

**Affiliations:** a Research Centre for Drugs and Diagnostics, Liverpool School of Tropical Medicine, Liverpool, UK; b Laboratorio Nacional de Salud Guatemala, Ministerio de Salud Publica y Asistencia Social de Guatemala, Villa Nueva, Guatemala; c QIAGEN Manchester Ltd, Skelton House, Lloyd Street North, Manchester, UK; d Universidad San Francisco de Quito, Quito, Ecuador

**Keywords:** Diagnostics, Chikungunya, Arboviruses, Molecular diagnostics,

## Abstract

Chikungunya was introduced into the Americas in 2015 causing a pandemic across the continent. Testing during the acute phase of infection relies on qRT-PCR, but available assays have a number of limitations. A qRT-PCR assay specific to the chikungunya E1 gene was designed using sequence data from contemporary strains. A probit analysis established the 95% limit of detection as 19.6 copies per reaction. We compared the assay with a US Centers for Disease Control (CDC) chikungunya qRT-PCR as the reference standard. The assay had a sensitivity and specificity of 98.4% and 100% in 90 samples retrospectively collected in Guatemala. In a further 74 febrile samples prospectively collected in Ecuador and Guatemala the test had a sensitivity and specificity of 100% and 98.4%, respectively. Sequencing the nsp4 gene of the discordant positive sample indicated the presence of chikungunya RNA, and mismatches to the primer binding sites of the CDC assay.

## 1 Introduction

Chikungunya virus (CHIKV), an alphavirus transmitted by *Aedes* mosquitoes, causes an acute febrile illness with a wide range of symptoms including fever, rash, and headache. Severe polyarthralgia occurs in up to 95% of cases (Weaver and Lecuit, 2015), which can progress to chronic polyarthritis in around 5% of patients (Ganu and Ganu, 2011).

In 2013, the Asian lineage of the CHIKV strain was reported in the island of St. Martin in the Americas. Initial reports were followed by a rapid spread across the Caribbean, causing over 790,000 cases.

Despite being a major pandemic (Cugola et al., 2016), only about 4% of the cases occurring in 2015 were confirmed by laboratory tests. The lack of confirmation hinders the accuracy of epidemiological data, as dengue (DENV) and Zika share the same vectors, are hyperendemic in the region and have similar clinical presentation.

Distinguishing acute DENV, Zika and CHIKV is important due to their potential complications. DENV can progress to severe dengue, characterized by hemorrhages and shock; Zika is associated with congenital and neurological anomalies which require an early diagnosis during pregnancy (Cao-Lormeau et al., n.d, Cugola et al., 2016), while CHIKV often leads to debilitating polyarthritis.

The methods available for CHIKV diagnosis depend on how many days the patient has been symptomatic. Enzyme Link Immuno-assays (ELISA) can detect Immunoglobulin (Ig) M antibodies after 5 days of symptoms and a raise of Ig G antibody titres can confirm the infections if paired samples are available (Prince et al., 2015). However ELISAs are unable to confirm infections between days 1–5 of symptoms (Yap et al., 2010).

CHIKV viraemia is present in the first 5–7 days of symptoms and RNA can be detected in serum or whole blood using molecular assays; usually using reverse transcriptase polymerase chain reaction (qRT-PCR). A number of qRT-PCR assays are commercially available, which are typically able to detect between 4 and 20 gene copies per reaction (Edwards et al., 2007, Panning et al., 2009, Pongsiri et al., 2012). Multiplexed assays to simultaneously detect CHIKV and DENV have also been designed (Cecilia et al., 2015, Waggoner et al., 2016) and a high throughput transcription-mediated amplification assay to screen asymptomatic blood donors is under development (Charles et al., 2015).

A frequently used qRT-PCR assay was developed by the US Centers of Disease Control and Prevention (CDC) in 2007 (Lanciotti et al., 2007), and is often considered the reference standard for CHIKV diagnosis. The assay was designed prior to the American pandemic, and therefore may not have incorporated contemporary strains, which could potentially reduce sensitivity.

We report here the design and validation of a qRT-PCR assay capable of detecting all lineages of CHIKV, which contains an internal control to identify sample inhibition. The assay is specific to a highly conserved region of the E1 gene and is suitable for laboratories capable of performing qRT-PCR.

## 2 Methods

### 2.1 Ethics statement

Samples obtained from Guatemala were anonymised samples submitted to the national reference surveillance laboratory (Laboratorio Nacional de Salud, LNS) for surveillance and diagnostic purposes. Samples were obtained with patients' consent and donated to the laboratories to conduct further testing and assay evaluation. The collection of samples from Esmeraldas, Ecuador was approved by the ethics committee of San Francisco de Quito University, Ecuador. The results of the study were anonymised and were not used for clinical management or surveillance purposes.

### 2.2 The study

A qRT-PCR was designed based upon contemporary CHIKV sequence data and underwent an analytical evaluation, followed by a field evaluation. We evaluated its limits of detection (LOD), linearity and reproducibility using RNA extracted from cultured virus, which was quantified via qRT-PCR. The assay was then compared to the CDC assay using a retrospective sample collection of 90 samples which were CHIKV CDC qRT-PCR positive and CHIKV-negative at the LNS in Guatemala and 74 prospectively collected febrile serum samples from Ecuador (n = 63) and Guatemala (n = 11).

### 2.3 Assay design

The primers and probe for the CHIKV E1 assay were designed to ensure maximum sequence complementarity with a wide range of strains from all geographical areas. Sequence alignments were carried out using ClustalW involving 100 geographically diverse CHIKV genomes and full and partial E1 gene sequences (North America [n = 4], Central America [n = 10], South America [n = 28], Caribbean [n = 17], Europe [n = 1], Africa [n = 15], Asia [n = 22], Oceania [n = 3],) retrieved from GenBank. The sequence alignments were carried out in the summer of 2015. Primer and probes were designed using Primer3, with binding sites selected to minimize the number of mismatches. Candidate primer and probe sets were checked for cross-reactivity using BLAST, and for secondary structure and self-complementarity using Mfold and OligoCalc. In adherence to MIQE guidelines (Bustin et al., 2011), we provide a PCR amplicon context sequence; the anchor nucleotide is at position 100,450, and the amplicon context sequence ranges from 100,390 and 100,523 (positions based on reference sequence NC 004612).

### 2.4 Viral culture

Viral stocks of the ECSA CHIKV Ross strain were produced using *Ae. albopictus* C6/36 cells infected at a multiplicity of infection (MOI) of 0.1 PFU (plaque forming units)/cell for 48 h at 27 °C. Stocks were constituted after two passages on C6/36 cells, titrated on Vero cells, and stored at -20 °C until use.

### 2.5 RNA extraction

RNA extractions were carried out from clinical samples and viral cultures using the QIAGEN Viral RNA extraction kit (QIAGEN, UK). The extractions required a 120 μl aliquot of the serum sample and eluted in 60 μl of elution buffer following the manufacturer's instructions.

### 2.6 CHIKV qRT-PCR assay

The CHIKV qRT-PCR assays were set up as follows, using reagents supplied with the QuantiFast Pathogen qRT-PCR + IC kit (QIAGEN, UK); 5 μl 5× QuantiFast pathogen master mix, 0.25 μl 100× QuantiFast Pathogen RT mix, 2.5 μl 10× internal control assay, 2.5 μl 10× internal control RNA, 0.4 μM E1 forward primer, 0.4 μM E1 reverse primer, 0.2 μM E1 probe, and 5 μl of template RNA or H_2_O no-template control in a total volume of 25 μ. The reactions were as follows; activation of reverse transcription at 50 °C for 20 minutes, followed by 95 °C for 5 minutes, and then 45 cycles of denaturation at 95 °C for 15 seconds, and extension at 60 °C for 30 seconds.

The CHIKV qRT-PCR assays were analyzed using a Rotor-gene Q system (QIAGEN, UK). A cut-off value of Ct 37 was chosen to allow direct comparison with the CDC reference assay, which utilizes this cut off. The threshold level was determined as being 0.13 during the investigation of assay reproducibility, as described below.

### 2.7 CDC qRT-PCR reference assay

We used a CHIKV qRT-PCR designed by the Diagnostic and Reference Laboratory, Arbovirus Diseases Branch, CDC, using the 6856F/6981c/6919-FAM primer set for the nsp4 gene as the reference assay (Lanciotti et al., 2007). The PCR reactions contained 12.5 μl Invitrogen SuperScript iii one-step 2× reaction mix, 0.5 μl SuperScript iii RT/Platinum Taq enzyme mix, 0.4 μM of each primer, 0.2 μM 6919-FAM probe, and 5 μl of template RNA. Sufficient molecular grade water was then added to produce a final reaction volume of 25 μl. The reactions were monitored using a Rotorgene Q (QIAGEN, UK).

The reaction parameters were as follows; reverse transcription at 50 °C for 30 minutes, followed by 95 °C for 2 minutes, and then 45 cycles of denaturation at 95 °C for 15 seconds, and extension at 60 °C for 30 seconds.

### 2.8 Plasmid generation and quantitation

A DNA plasmid containing the CHIKV amplicon was used as a quantifiable stock of the assay target, for determining CHIKV RNA concentration via a standard curve. A 3817 bp double stranded DNA plasmid containing the CHIKV assay amplicon was obtained commercially from Oxford Genetics Ltd. (UK). The plasmid concentration was quantified using a Qubit 3.0 fluorometer (ThermoFisher, UK).

### 2.9 Sequencing

Serum samples (n = 8) were selected for sequencing of both the nsp4 and E1 genes, to examine for variation in the primer binding sites of both the index and reference tests. The samples included one with discordant test results from Guatemala, and also samples with concordant results sourced from Guatemala (n = 5) and Ecuador (n = 2). All samples chosen were <Ct 30 in order to ensure sufficient RNA for the sequencing methodology. Reverse transcription was conducted using the Superscript IV reverse strand synthesis system (Invitrogen, UK), following the manufacturer's instructions. The resulting cDNA from each sample was amplified by PCR using the E1 and nsp4 primer sets, using the proof reading Phusion high fidelity DNA polymerase reaction mix (Thermo, UK), and 0.4 μM of forward and reverse primers. Primer sequences for amplification of a 1320 bp fragment of the E1 gene (positions 9991–11,310) were taken from (Shrinet et al., 2012) (E1-gene-F and E1-gene-R primers). Primers for the amplification of a 763 bp region of the nsp4 gene (6732F and 7495R) were taken from (Schuffenecker et al., 2006). Cycling parameters for PCR reactions containing both sets of primers were as described (Nunes et al., 2015).

PCR amplicons were purified using a QIAquick PCR purification kit (QIAGEN, UK), following the manufacturer's instructions. The forward and reverse strand of purified amplicons were sequenced commercially at Source Bioscience (UK), using the same primers.

### 2.10 Assay characteristics

The linearity of the assay was determined by testing eight sequential 1:10 dilutions of CHIKV RNA, in five replicates. A line of best fit was fitted via regression analysis on a plot of the Ct value (*y*-axis) and log concentration (*x*-axis). The portion of the line that maintained an R^2^ value of over 0.99 was regarded as indicating the linear range of the assay.

### 2.11 Reproducibility

The inter and intra-assay coefficient of variation (CV) was calculated as a measure of reproducibility and was determined using three 1:100 dilutions of CHIKV RNA. Concentrations of 1 × 10^7^, 1 × 10^5^ and 1 × 10^3^ per reaction were chosen to represent a high, medium and low quantity of RNA over the linear range of the assay. Each dilution was assayed using the CHIKV qRT-PCR in triplicate, and the experiment was repeated five times over consecutive days by the same operator, using the same batch of reagents. Average baseline and maximum fluorescence values were collected from these experiments and averaged to determine the Ct calling. The threshold was set at the value of the baseline fluorescence plus 10% of the difference between the baseline and maximum fluorescence. This value was determined to be 0.13, and this level was used for all assays, including previous assays, which were reanalysed accordingly.

### 2.12 LOD

The LOD was determined by testing RNA dilutions of a known concentration. Viral RNA extracted from culture was quantified via a standard curve consisting of dilutions of the amplicon containing plasmid. This RNA was then diluted to give 1 × 10^8^ copies per reaction, and diluted sequentially to a concentration giving 1 copy per reaction. A total of five reactions were conducted per dilution. Further experiments were conducted testing 100, 75, 50, 25, 15 and 10 copies, with five reactions per dilution. Data was analyzed using a probit regression analysis to calculate the 95% and 50% LOD.

### 2.13 Specificity

The CHIKV qRT-PCR was challenged with 0.1 μg of genomic RNA for DENV serotypes 1 (Strain TC974), 2 (Strain R062), 3 (Strain H87), 4 (Strain TC1000), Influenza A H3N2 virus (Strain A/Wuhan/359/95), Influenza B (Strain B/Brisbane), measles (Strain Mvs/London.GBR/25.07), Yellow fever (French Neurotropic strain), Mayaro virus (Strain TC652) and Zika virus (Brazilian epidemic strain). Additionally the assay was tested using 48 RNA samples submitted to LNS in 2015 confirmed as being positive for DENV using the CDC DENV 1–4 qRT-PCR assay (26), and negative by the CDC CHIKV qRT-PCR assay. The samples included DENV1 (3), DENV2 (38), DENV3 (3) and DENV4 (4).

### 2.14 Bank sample collection

Stored serum samples kept at −80 °C for between 1 and 4 weeks at the LNS were used as alpha evaluation samples in clinical specimens. These included 64 CDC CHIKV PCR-positive and 26 CDC CHIKV PCR-negative samples. After RNA extraction, all samples were tested in the UK using the CDC CHIKV and our CHIKV qRT-PCR assays using the Rotorgene Q.

### 2.15 Prospective sample collection

Serum samples were obtained from 63 patients presenting at a fever clinic in Hospital Delfina Torres Conchan in Esmeraldas, Ecuador, in July 2015 with symptoms <10 days duration. All eligible patients presenting over 3 days were included in the study. Of these, 10 patients had symptoms for <1 day; 16 for 2 days, 13 for 3 days, 6 for 4 days, 10 for 5 days, 1 for 6 days and 2 for 7 days. Their median age was 20 years with a range from 6 months to 81 years. Eleven additional serum samples acquired prospectively from patients with febrile illness were obtained by the LNS, Guatemala. Samples were frozen at −20 °C, stored for a maximum of 1 week and then stored at −80 °C. Samples were then transported to the UK for further testing. RNA samples were tested on the same day with both assays and technicians were blinded to the results of other tests to avoid bias.

## 3 Results

### 3.1 Assay characteristics and reproducibility

**Fig. 1:**
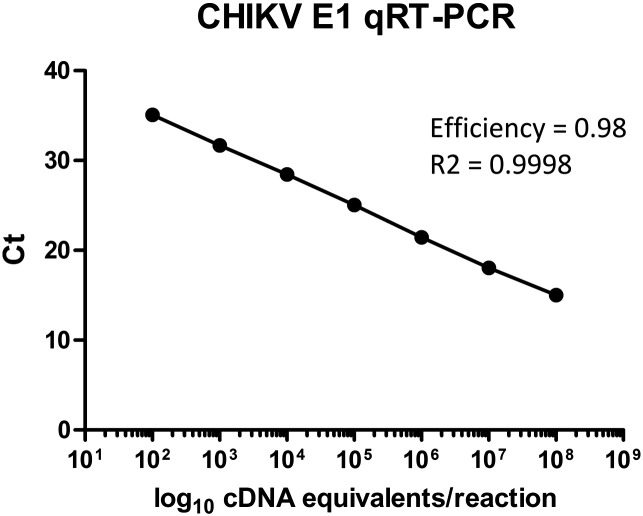
Linear range of the CHIKV E1 RT-PCR assay.

The linear range of the assay was 1 × 10^2^ to 1 × 10^8^ copies per reaction, with an R^2^ value of 0.9998 over this range (Fig. 1). The efficiency of the qRT-PCR was 0.98. The inter and intra assay coefficient of variation (CV) and average efficiency are shown in Table 1. The inter and intra assay CV were 0.73% and 0.38%, respectively. The mean (SD) efficiency of the PCR across the 5 experiments was 0.956 (0.02).

**Table 1:** Average Ct, SD, and efficiency values are the average of 5 experiments containing 3 replicates. SD, standard deviation; CV, coefficient of variation.

	cDNA equivalents/reaction		
	1 × 10^7^	1 × 10^5^	1 × 10^3^
Average Ct	18.13133	24.958	31.86866667
Average SD	0.211689	0.106449	0.190857713
Inter assay CV	1.17%	0.43%	0.60%
Intra assay CV	1.15%	0.28%	0.46%


	Overall results
Overall inter assay CV	0.73%
Overall intra assay CV	0.38%
Range of efficiency	0.93–0.98 (mean = 0.956)

### 3.2 Limit of detection

The 95% LOD of the qRT-PCR was equivalent to 19.6 (95% CI: 14.3–29.6) copies of the target gene per assay, equating to 1.96 × 10^3^ viral genomes per ml of serum. The 50% LOD was 10.6 (95% CI: 7.9–20.7) copies of the target gene per reaction. If a cut off of Ct 40 was used, the sensitivity increased and the 95% and 50% LOD were 17.7 (95% CI: 13.2–27.8) and 7.1 (95% CI: 5.2–16.6) copies of the target gene respectively, with no apparent effect on specificity.

### 3.3 Specificity

The addition of non-target viral RNA did not result in a detectable signal in the CHIKV qRT-PCR assay. Amplification did not occur when the 48 DENV-positive samples were tested. The primer and probe sequences were compared with sequences in the NCBI nucleotide database and no non-target matches were found.

### 3.4 Bank sample collection

The CHIKV qRT-PCR was positive in 63/64 of the samples positive by the CDC qRT-PCR assay (sensitivity 98.4% [95% CI: 91.6–99.96%]) (Table 2). CHIKV amplification was detected in the negative sample, at a Ct 42. The Ct value obtained by the CDC assay was 36.52.

**Table 2:** Diagnostic accuracy of the CHIKV qRT-PCR against the CDC qRT-PCR in bank and prospective specimens.

CHIKV E1 qRT-PCR	CDC CHIKV qRT-PCR	
Bank samples	**Positive (64)**	**Negative (26)**
Positive	63	0
Negative	1	26
Prospective samples	**Positive (10)**	**Negative (64)**
Positive	10	1
Negative	0	63

All 26 CDC qRT-PCR-negative samples were negative by the CHIKV assay (specificity 100% [95% CI 86.77–100%]), with an overall agreement of 98.9%.

### 3.5 Prospective samples

Three (27%) of 11 Guatemalan and 7 (11%) of 63 Ecuadorian prospective samples were CDC qRT-PCR positive. The CHIKV qRT-PCR assay identified 10/10 of the samples positive by the reference assay (sensitivity 100%, 95% CI: 69–100%, Table 2). One of 64 CDC qRT-PCR-negative samples was positive by the CHIKV qRT-PCR (specificity 98.4%, 95% CI: 92–99.9%), with an overall agreement of 98.6%. This discordant sample had a Ct value of 18 with the index assay and no detectable amplification with the CDC qRT-PCR.

### 3.6 Sequencing

Sequencing of the nsp4 gene of the discordant sample solely positive by the index test identified two mismatches in the target region of the forward CDC primer at positions 8 and 17 (5′ > 3′, GenBank accession number KX296741). A further 5 randomly selected samples (ct <30) from Guatemala were also sequenced, and the same base changes were found to be present in each sample. Additionally, the nsp4 gene of two samples from Ecuador were sequenced, and were both found to carry one of the two mismatches, at position 8 of the forward primer. The E1 gene of the above isolates was also sequenced (example: KX296738), and no strains were found to have mismatches in the E1 primer or probe binding sites.

### 3.7 Internal control performance

There were no failures in the internal control in any of the 164 samples tested, allowing all samples to be included in the study. Mean (SD) Ct values were 25.6 (0.53).

## 4 Discussion

The aim of this study was to design and perform a small evaluation of a qRT-PCR assay capable of detecting all circulating lineages of CHIKV. Central to this was the use of contemporary sequence data to identify primer and probe binding sites that are highly conserved across all CHIKV lineages. The ability to identify all CHIKV lineages is important as returning travelers can instigate CHIKV outbreaks of strains from diverse geographical areas. Brazil, for example, reported outbreaks of the Asian lineage, introduced via the Caribbean outbreak, and of the East African strain, which is thought to have been introduced via a traveler from Angola (Nunes et al., 2015).

The 95% Limit of Detection of our assay was 19.6 gene copies per reaction, which, when combined with the QIAGEN Viral RNA extraction kit, and assuming 100% efficiency in each step of the process, equates to 1.96 × 10^3^ genome copies/ml of sample. This is over a hundred fold lower than the mean viraemia of 5.6 × 10^5^ and 3.4 × 10^3^ plaque forming units (PFU)/ml reported in laboratory confirmed symptomatic and asymptomatic CHIKV cases in Thailand (Appassakij et al., 2013). The LOD of our assay lies over one log10 below the range of the viral titres reported in the Caribbean (Charles et al., 2015, Gallian et al., 2014) and is comparable to reported CHIKV qRT-PCR assays reporting LODs ranging from 5.3 to 27 RNA copies per reaction (Santiago et al., 2013).

The linear range of the assay spanned 10^2^ to 10^8^ copies of viral RNA per reaction, with an R2 value of 0.9998 indicating excellent linearity. The assay was also highly reproducible. These factors allow the use of the assay as a quantitative tool (Peixoto et al., 2004), when paired with a standard curve of known target concentration.

The single discordant sample among the retrospective samples had a Ct of 36.5 in the reference assay, whilst our assay amplified the RNA over the Ct 37 threshold. The mean (SD) Ct values obtained using the CDC assay were lower (22.41, SD 6.67) than our CHIKV assay (24.34, SD 6.67), possibly due to the inclusion of an internal control assay reducing amplification efficiency, or differences in the reaction mix reagents used for the two assays.

The index PCR reported a single CDC PCR-negative sample as positive, resulting in a specificity of 98.4% (95% CI: 92–99.9%). This sample had a particularly low Ct, indicating a copy number of 2.2 × 10^7^ copies per reaction, equating to 2.2 × 10^9^ viral genomes per ml. The detection of two separate CHIKV genes through sequencing indicates that CHIKV RNA was likely present in the sample, and this positive result is unlikely to be due to lack of specificity. The sequence of the target of reference PCR included two mismatches in the forward primer of this assay, which could potentially have led to the failure to amplify. However, sequencing of five reference PCR positive samples from Guatemala also found these same mismatches in all samples, indicating that they alone do not prevent PCR amplification. The Ct values obtained for the CDC assay were lower (mean −0.96) than those obtained using the E1 assay, demonstrating no apparent loss in amplification efficiency due to the mismatches. Subsequent analysis of this region in sequence data submitted to GenBank showed these two mutations to be present in the majority of Asian CHIKV strains introduced into the Americas via the Caribbean outbreak. Example sequences include KY703993.1 (Nicaragua, 2014), KY680370.1 (USA, 2014) KT327165.2 (Mexico 2014) KR559495.1 (Puerto Rico, 2014) and Brazil (KP164567.1). Notably these mutations were absent in the ECSA origin strains that were introduced into Brazil in 2014 (KP164570.1).

Mutations in primer binding sites are an inevitable issue in the use of molecular diagnostics, especially in highly mutable RNA viruses. These mutations can cause lower binding efficiency, resulting in reduced sensitivity and inaccurate quantification and possibly result in false negative results, as reported for a diverse range of viral targets, including Eastern Equine Encephalitis, herpes simplex and avian influenza (Armstrong et al., 2012). Frequent update of assays via the incorporation of contemporary strains in the design process is necessary to negate this variation.

The validation of the assay was carried out using samples collected in Guatemala and Ecuador in July and August 2015, which solely included strains descended from the Asian strain responsible for the Central and South American outbreak. The RNA used during the assay development was obtained from cultivated ESCA strain CHIKV; however, no clinical ESCA, West African or Asia-circulating Asian strains were tested, and further evaluation would be required to determine the performance of the assay with these strains.

The study included a small scale clinical evaluation, and further work including a greater number of more diverse samples is required to evaluate the performance of the assay in more detail.

## 5 Conclusion

We describe the design and evaluation of a Taqman based real-time qRT-PCR assay for the detection of CHIKV via the amplification of an 110 bp fragment of the E1 gene. The assay has comparable sensitivity and specificity to the CDC CHIKV qRT-PCR. The 95% and 50% LOD were estimated as 19.6 and 10.6 copies per reaction, respectively.

The qRT-PCR assay detailed here has undergone a robust analytical evaluation to determine the most important aspects of assay performance to provide a high level of confidence in its application for diagnosing acute CHIKV infection. The assay has been designed to detect all lineages of CHIKV, with particular focus on the strains causing the current outbreaks in South America. The assay evaluation has been reported according to STARD 2015 reporting guideline for diagnostic accuracy studies, as supported by the supplementary STARD checklist.

## Appendix A Supplementary data

Supplementary material. STARD checklist Image 1
